# The Presence of Periodontitis Exacerbates Non-Alcoholic Fatty Liver Disease via Sphingolipid Metabolism-Associated Insulin Resistance and Hepatic Inflammation in Mice with Metabolic Syndrome

**DOI:** 10.3390/ijms24098322

**Published:** 2023-05-05

**Authors:** Zhongyang Lu, Yanchun Li, Nityananda Chowdhury, Hong Yu, Wing-Kin Syn, Maria Lopes-Virella, Özlem Yilmaz, Yan Huang

**Affiliations:** 1 Division of Endocrinology, Diabetes and Metabolic Diseases, Department of Medicine, Medical University of South Carolina, Charleston, SC 29425, USA; 2 Department of Oral Health Sciences, The James B. Edwards College of Dental Medicine, Medical University of South Carolina, Charleston, SC 29425, USA; 3 Division of Gastroenterology and Hepatology, Saint Louis University School of Medicine, Saint Louis, MI 63110, USA; 4 Division of Gastroenterology and Hepatology, Medical University of South Carolina, Charleston, SC 29425, USA; 5 Department of Physiology, Faculty of Medicine and Nursing, University of the Basque Country, Universidad del Pa S Vasco/Euskal Herriko Univertsitatea (UPV/EHU), 48940 Leioa, Spain; 6 Ralph H. Johnson Veterans Affairs Medical Center, Charleston, SC 29401, USA

**Keywords:** metabolic syndrome, periodontitis, non-alcoholic fatty liver disease, imipramine, acid sphingomyelinase, inflammation

## Abstract

Clinical studies have shown that periodontitis is associated with non-alcoholic fatty liver disease (NAFLD). However, it remains unclear if periodontitis contributes to the progression of NAFLD. In this study, we generated a mouse model with high-fat diet (HFD)-induced metabolic syndrome (MetS) and NAFLD and oral *P. gingivalis* inoculation-induced periodontitis. Results showed that the presence of periodontitis increased insulin resistance and hepatic inflammation and exacerbated the progression of NAFLD. To determine the role of sphingolipid metabolism in the association between NAFLD and periodontitis, we also treated mice with imipramine, an inhibitor of acid sphingomyelinase (ASMase), and demonstrated that imipramine treatment significantly alleviated insulin resistance and hepatic inflammation, and improved NAFLD. Studies performed in vitro showed that lipopolysaccharide (LPS) and palmitic acid (PA), a major saturated fatty acid associated with MetS and NAFLD, synergistically increased the production of ceramide, a bioactive sphingolipid involved in NAFLD progression in macrophages but imipramine effectively reversed the ceramide production stimulated by LPS and PA. Taken together, this study showed for the first time that the presence of periodontitis contributed to the progression of NAFLD, likely due to alterations in sphingolipid metabolism that led to exacerbated insulin resistance and hepatic inflammation. This study also showed that targeting ASMase with imipramine improves NAFLD by reducing insulin resistance and hepatic inflammation.

## 1. Introduction

Periodontitis is primarily a bacterial infection of the supporting structures of teeth, characterized by tissue inflammation and destruction that eventually lead to tooth loss [1,2]. The interplay between periodontal pathogens and the host inflammatory response is critical to determine susceptibility to inflammation and the severity of periodontitis [3,4,5,6]. During periodontitis, oral pathogens stimulate the host’s innate immune system to upregulate proinflammatory mediators, such as inflammatory cytokines, that play a pivotal role in the progression of periodontitis [7]. Since type 2 diabetes mellitus (T2DM) and metabolic syndrome (MetS), a pre-diabetic state [8], coinciding with a chronic low-level host systemic inflammation [9,10], patients with T2DM or MetS have a higher risk of developing periodontitis as compared to patients without T2DM or MetS [3,11,12,13]. Therefore, periodontitis is considered a complication of T2DM or MetS [3,11,12].

A large number of studies have shown that the consequences of periodontitis extend far beyond oral health and are positively associated with many major diseases, such as atherosclerosis [14], stroke [15], osteoporosis [16], Alzheimer’s disease [17,18,19,20], orodigestive cancers and non-alcoholic fatty liver disease (NAFLD) [21,22,23]. NAFLD is characterized by the accumulation of triglycerides and other fats (steatosis) in the liver in the absence of alcoholic consumption [24]. Similar to periodontitis, NAFLD is also a complication of T2DM and MetS [25]. NAFLD progresses from hepatic steatosis to non-alcoholic steatohepatitis (NASH) in 20–30% of these cases, which can further progress to cirrhosis or liver cancer [25,26]. NASH, an advanced stage of NAFLD, is characterized by hepatic steatosis, inflammation, hepatocellular injury, and fibrosis [27]. 

A number of studies in recent years have shown a positive association between periodontitis and NAFLD [21,22,23,28,29]. Among them, Weintraub et al. analyzed the data from the National Health and Nutrition Examination Survey (NHANES) and found that NAFLD was associated with tooth loss and periodontitis [30]. Kim et al. used fatty liver index (FLI), a non-invasive surrogate marker and predictor of NAFLD, to determine whether FLI is associated with periodontitis. They found that higher FLI was associated with a higher prevalence of periodontitis after adjusting for confounding factors [31]. A few studies, however, failed to show an association between periodontitis and NAFLD [32,33]. For example, Wijarnpreecha et al. conducted a meta-analysis that showed a significant association between NAFLD and periodontitis. However, the association lost its significance when adjusted for metabolic parameters such as insulin resistance [32], suggesting that those metabolic conditions are the predisposing factors for NAFLD. Given that both periodontitis and NAFLD are associated with T2DM and MetS, the above findings suggest that periodontitis may be associated with NAFLD progression simply by worsening T2DM- or MetS-related metabolic parameters such as insulin resistance. Overall, the conflicting results strongly suggest that more studies are required to elucidate the mechanisms underlying the interaction of periodontitis with NAFLD.

In previous studies designed to understand the mechanisms involved in the interaction of periodontitis with NAFLD, it has been shown that periodontitis-related dysregulation of the oral microbiome leads to the dissemination of oral pathogens and bacterial byproducts such as lipopolysaccharide (LPS) to the circulation and liver, therefore promoting increased systemic and hepatic inflammation [3,34]. In agreement with the above reports, our study has demonstrated that LPS has a synergistic interaction with palmitic acid (PA), a major saturated fatty acid (SFA) associated with dyslipidemia in MetS and NAFLD [35], and that interaction leads to a strong upregulation of proinflammatory genes in macrophages [36]. Since it is well known that macrophages play a key role in hepatic inflammation and NAFLD progression [37], the synergy between LPS and PA on the upregulation of proinflammatory genes in macrophages represents a novel immuno-metabolic crosstalk potentially involved in the interaction of periodontitis with NASH. 

Our studies exploring the molecular mechanisms involved in the crosstalk between LPS and PA have shown that dysregulation of sphingolipid metabolism and increased production of some bioactive sphingolipids such as ceramide and sphingosine 1-phosphate (S1P) played a pivotal role [36,38]. Specifically, we have shown that in macrophages, LPS and PA synergistically stimulate acid sphingomyelinase (ASMase), thus leading to increased hydrolysis of sphingomyelin and increased cellular ceramide content [36]. The increase in cellular ceramide led to the upregulation of proinflammatory genes in macrophages [36,38]. We have also shown that inhibiting ASMase reduced the cellular content of ceramides in macrophages and improved both NAFLD and periodontitis in animal models [39,40]. However, it remains unclear if ASMase-related dysregulation of sphingolipid metabolism is involved in the interaction of periodontitis with NAFLD progression.

In the current study, we created a mouse model with both NAFLD induced by a high-fat diet (HFD) and periodontitis induced by oral *P. gingivalis* inoculation. The purpose of this study is to further explore the molecular mechanisms behind the interaction of periodontitis with NAFLD and to determine if the presence of periodontitis contributes to NAFLD, but targeting ASMase-related sphingolipid metabolism improves NAFLD.

## 2. Results

### 2.1. The Effect of HFD Feeding, P. gingivalis Inoculation and Imipramine on Blood Levels of Insulin, Glucose and Lipids and Hepatic Injury

**Figure 1:**
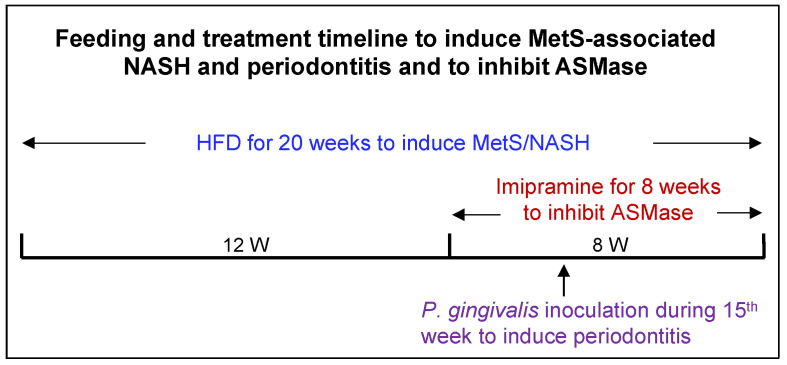
The timeline for induction of MetS-associated NAFLD and periodontitis and treatment with imipramine. Six weeks old male C57BL/6 mice were fed high-fat diet (HFD) for 20 weeks to induce metabolic syndrome (MetS) and MetS-associated nonalcoholic steatohepatitis (NASH). Starting from Week 13, part of the mice were treated with imipramine for eight weeks to inhibit acid sphingomyelinase (ASMase). Starting from Week 13, part of the mice were treated with antibiotics for seven days for the preparation of oral *P. gingivalis* inoculation. In Week 15, part of the mice received *P. gingivalis* inoculation to induce periodontitis. All mice were sacrificed at the end of 20 weeks.

**Figure 2:**
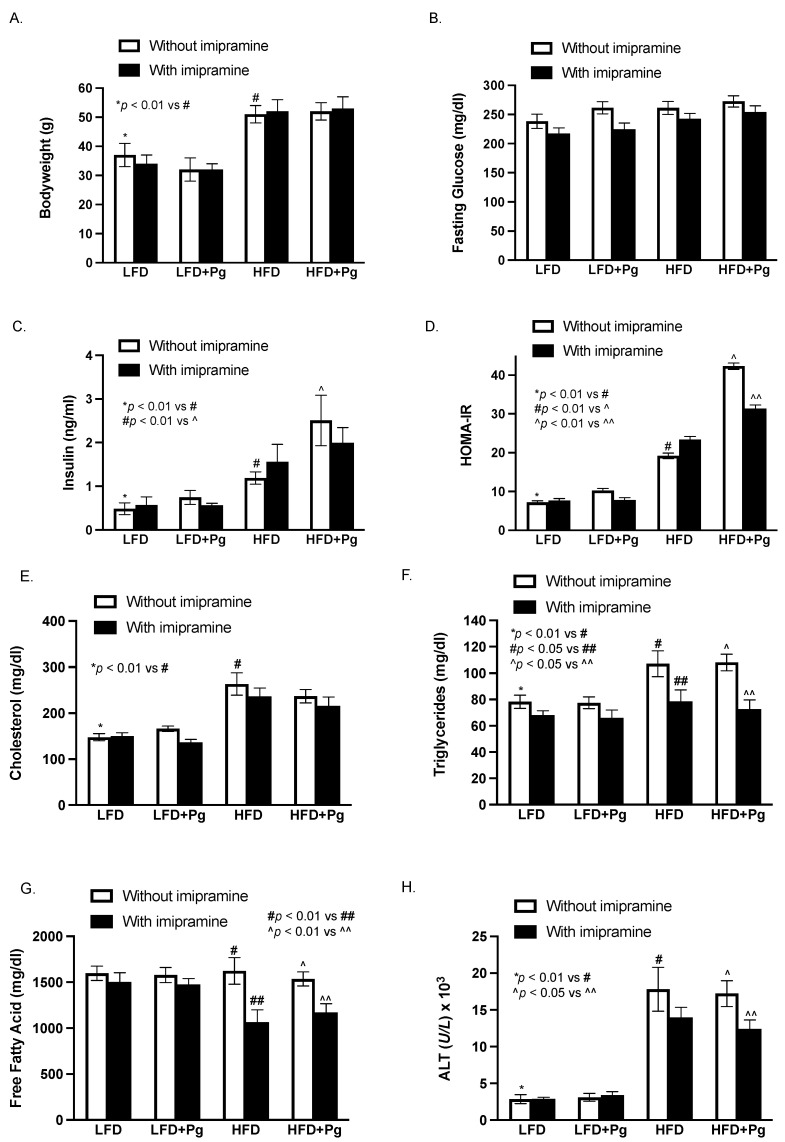
The effect of HFD feeding, oral *P. gingivalis* (Pg) inoculation, and imipramine treatment on the metabolic parameters and hepatic injury, including body weight (**A**), fasting glucose (**B**), insulin (**C**) and insulin resistance (HOMA-IR) (**D**), cholesterol (**E**), triglyceride (**F**), free fatty acid (**G**), and ALT (**H**) in mice. Six-week-old male C57BL/6 mice were fed HFD for 20 weeks to induce MetS and MetS-associated NAFLD and inoculated orally with Pg in the 15th week to induce periodontitis. Part of the mice were also treated with imipramine in the last eight weeks. Control mice were fed LFD and inoculated orally with a vehicle of Pg. At the end of the feeding and treatments, blood was collected, and the serum levels of the metabolic parameters were quantified. The data are mean ± SD (*n* = 12).

As shown in Figure 1, mice were fed HFD to induce MetS and MetS-associated NAFLD and orally inoculated with *P. gingivalis* to induce periodontitis. Part of the mice were treated with imipramine, a well-established inhibitor of ASMase [40,41]. At the end of the study, the effects of HFD feeding, *P. gingivalis* inoculation, and imipramine on metabolic parameters and hepatic injury were determined. As shown in Figure 2, HFD feeding for 20 weeks induced MetS by increasing body weight, insulin levels, homeostasis model assessment of insulin resistance (HOMA-IR), and lipid levels, including cholesterol and triglycerides as compared to low-fat diet (LFD) feeding. Although HFD increased insulin levels, it did not reduce fasting glucose, indicating the presence of insulin resistance. HFD feeding also markedly increased serum alanine transaminase (ALT), suggesting that HFD induced hepatic injury. The oral *P. gingivalis* inoculation alone had no significant effect on metabolic parameters in mice fed LFD but further increased HFD-induced insulin secretion and insulin resistance. In contrast, imipramine treatment reduced insulin resistance, triglycerides, free fatty acids (FFAs), and ALT in mice fed HFD, suggesting that imipramine treatment improves MetS and MetS-related hepatic injury.

### 2.2. P. gingivalis Inoculation and HFD Feeding Promote, but Imipramine Alleviates Alveolar Bone Loss

**Figure 3:**
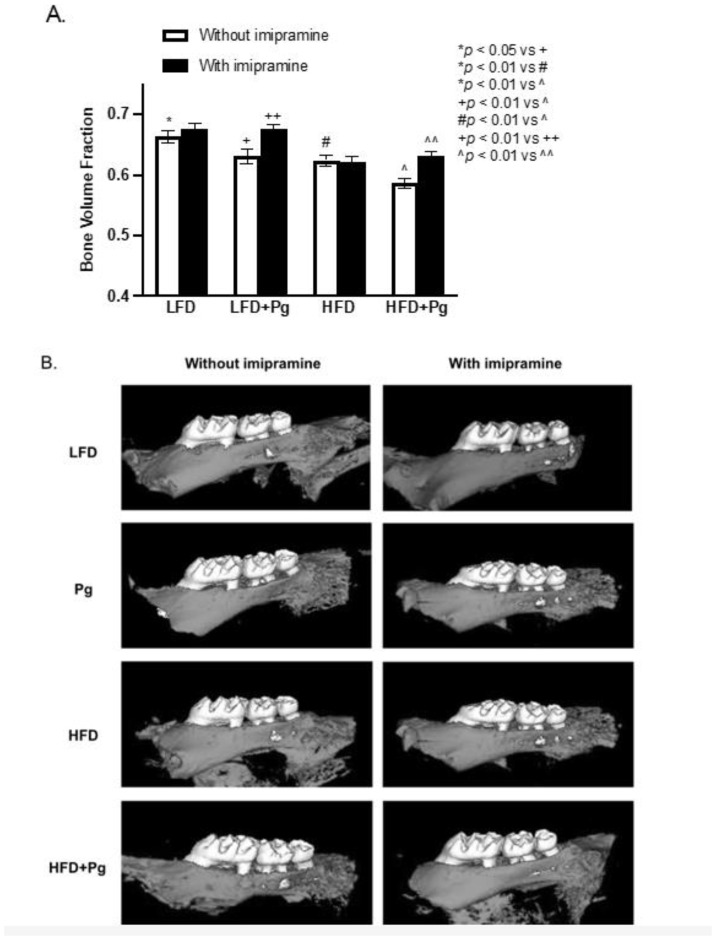

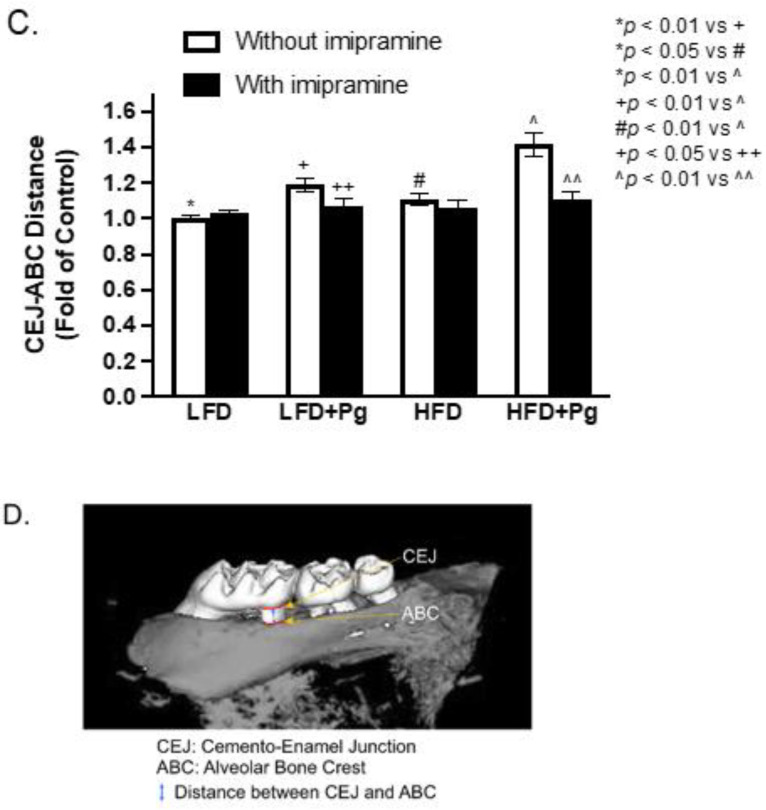
The effect of HFD feeding, oral *P. gingivalis* (Pg) inoculation, and imipramine treatment on alveolar bone loss. C57BL/6 mice were fed HFD for 20 weeks to induce MetS and NAFLD and inoculated orally with Pg in the 15th week to induce periodontitis. Part of the mice were also treated with imipramine in the last eight weeks. After the feeding and treatments, the maxillae were scanned with micro-computed tomography (mCT), and the bone volume fraction was determined (**A**). In addition, the liner distances from CEJ to ABC for the first and second molars were also measured. Representative mCT images for eight groups were shown (**B**), and the CEJ-ABC distances were presented as folds of the LFD-fed control mice (**C**). The locations of the CEJ and ABC and the distance between CEJ and ABC were shown (**D**). The data are mean ± SD (*n* = 12).

We performed micro-computed tomography (μCT) scanning on maxillae to quantify bone volume fraction (BVF). Results (Figure 3 and Table 1) showed that oral *P. gingivalis* inoculation and HFD feeding significantly induced alveolar bone loss by 4.90% and 5.88%, respectively, and the combination of *P. gingivalis* inoculation and HFD feeding further increased alveolar bone loss by 11.46%, revealing a cooperative effect of *P. gingivalis* inoculation and HFD feeding on alveolar bone loss. In contrast, imipramine treatment not only prevented bone loss but significantly increased the BVF by 8.47% in mice treated with *P. gingivalis* inoculation alone and by 7.50% in mice fed HFD and inoculated with *P. gingivalis*. In addition, we also measured the distance from the cemento–enamel junction (CEJ) to the alveolar bone crest (ABC) as previously described [42]. Results showed that *P. gingivalis* inoculation or HFD plus *P. gingivalis* inoculation significantly increased CEJ-ABC distance, but imipramine treatment reduced CEJ-ABC distance (Figure 3B–D).

### 2.3. P. gingivalis Inoculation and HFD Feeding Stimulate, but Imipramine Inhibits Periodontal Inflammation and Bone Resorption

**Figure 4:**
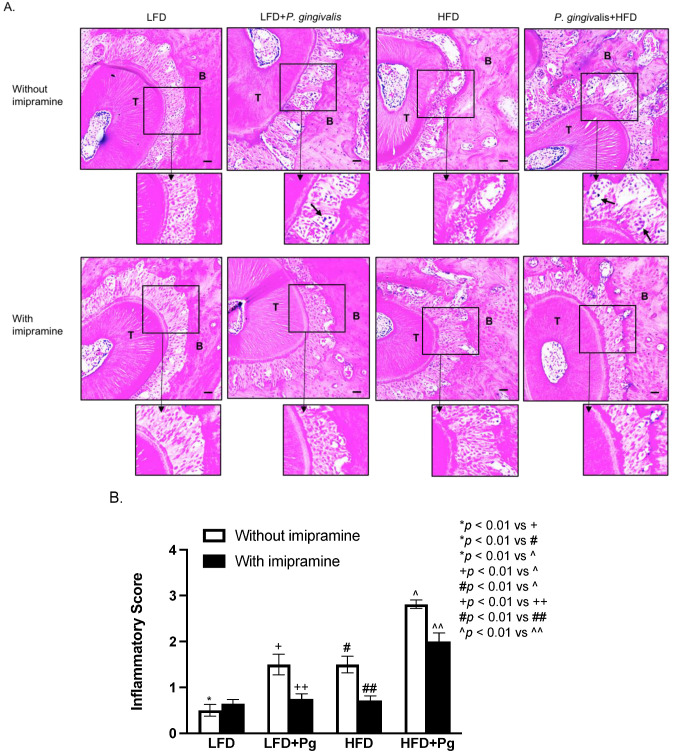
Histological analysis of periodontal tissues of mice after HFD feeding, oral *P. gingivalis* inoculation, and imipramine treatment. After performing the mCT of the maxillae as described above, the tissues were sectioned and stained with hematoxylin and eosin (H/E) for histological evaluation of leukocyte infiltration and bone resorption. (**A**) The images of the representative tissue sections with H/E staining focus on the area of the periodontal ligament, teeth, and alveolar bones. The images in the insets were enlarged, and the arrows indicated infiltrated leukocytes. Scale bar = 100 μm. (**B**) The inflammatory scores were made according to the degree of leukocyte infiltration and bone resorption as described in Materials and Methods. The data are mean ± SD (*n* = 12). T: tooth; B: alveolar bone.

Histological analysis of maxillae was conducted to determine the effects of *P. gingivalis* inoculation, HFD feeding, and imipramine treatment on periodontal inflammation and bone resorption. We assessed the leukocyte infiltration in the periodontal ligaments and bone resorption using a scoring system as described previously [43], and our results showed that either *P. gingivalis* inoculation or HFD feeding increased inflammatory score, but the combination of *P. gingivalis* inoculation and HFD feeding led to a cumulative increase of the score (Figure 4A,B). However, imipramine significantly reduced inflammatory scores in mice with *P. gingivalis* inoculation and *P. gingivalis* inoculation plus HFD feeding (Figure 4A,B). 

### 2.4. HFD Feeding or HFD Feeding plus P. gingivalis Inoculation Promotes, but Imipramine Ameliorates Hepatic Steatosis

**Figure 5:**
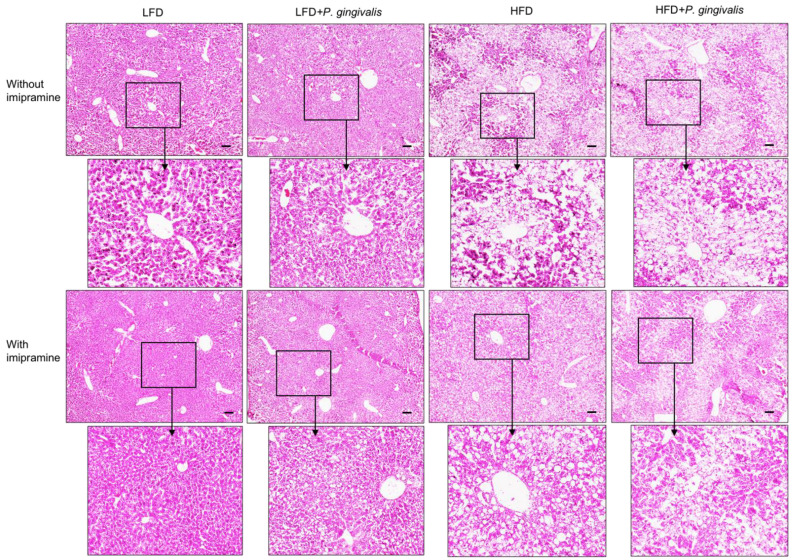
Histological analysis of liver tissue of mice after HFD feeding, oral *P. gingivalis* (Pg) inoculation, and imipramine treatment. C57BL/6 mice were fed HFD to induce MetS/NAFLD and orally inoculated with Pg to induce periodontitis. Part of the mice were also treated with imipramine. At the end of the study, the livers of the mice were dissected, sectioned, and stained with hematoxylin and eosin for histological analysis. Representative images of liver sections in eight groups were shown, and the images in the insets were enlarged. Scale bar = 100 μm.

**Figure 6:**
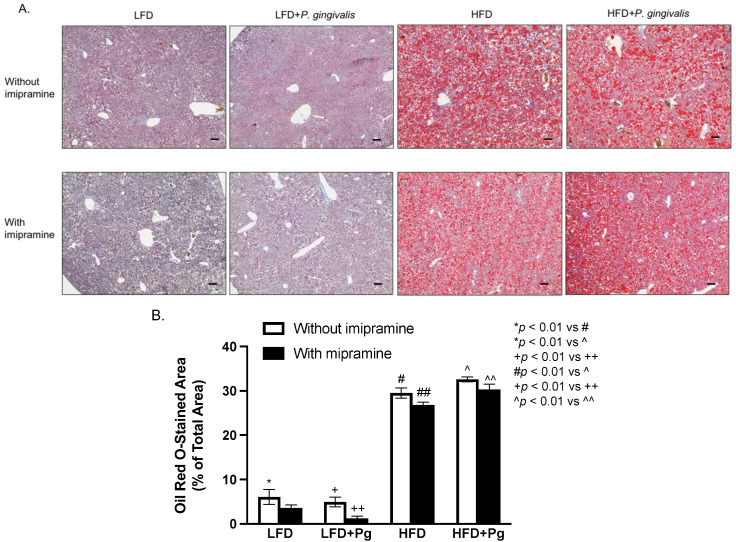
The effect of HFD feeding, oral *P. gingivalis* (Pg) inoculation, and imipramine treatment on hepatic steatosis. C57BL/6 mice were fed HFD to induce MetS/NAFLD and orally inoculated with Pg to induce periodontitis. Part of the mice were also treated with imipramine. At the end of the study, the livers of the mice were dissected, sectioned, and stained with Oil Red O. (**A**) Representative images of liver sections with Oil Red O staining. (**B**) The positively stained areas of Oil Red O staining in tissue sections were quantified and compared among all groups. Scale bar = 100 μm. The data are presented as means ± SD (*n* = 12).

To elucidate how periodontitis affects NAFLD, we performed a histological analysis of liver tissue. Results from hematoxylin and eosin (H&E) staining (Figure 5) showed that while *P. gingivalis* inoculation alone had no effect on fat accumulation in the liver, HFD feeding, or HFD feeding, plus *P. gingivalis* inoculation-induced fat accumulation. In contrast, imipramine treatment reduced the liver fat content. To quantify the amount of fat in the liver, we performed Oil Red O staining on liver tissue sections. Results (Figure 6A,B) showed that HFD feeding markedly increased fat accumulation, and the combination of HFD feeding and *P. gingivalis* inoculation further increased it. However, imipramine treatment moderately ameliorated hepatic steatosis induced by HFD feeding or by HFD feeding plus *P. gingivalis* inoculation. 

### 2.5. P. gingivalis Inoculation and HFD Feeding Stimulate, but Imipramine Inhibits Hepatic Inflammation

**Figure 7:**
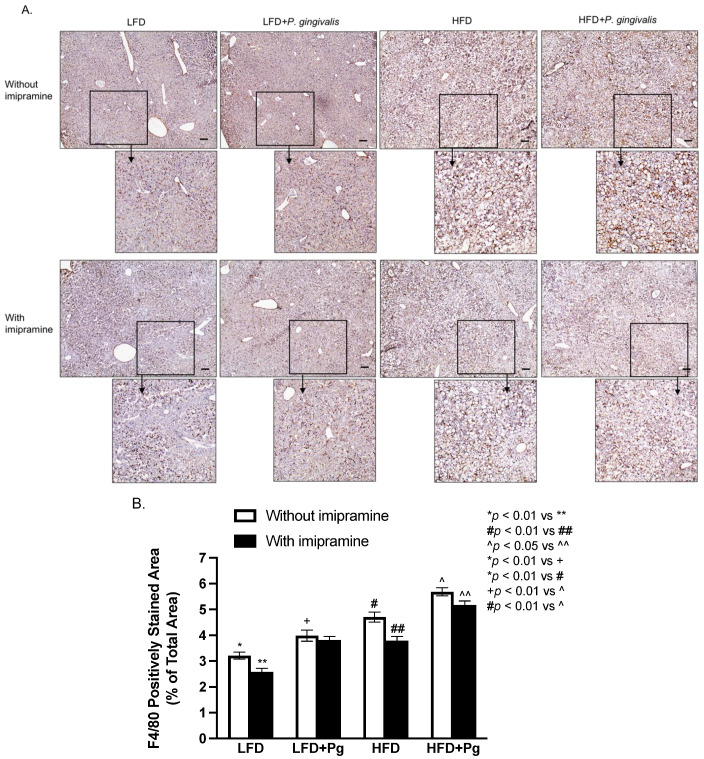
The effect of HFD feeding, oral *P. gingivalis* (Pg) inoculation, and imipramine treatment on hepatic inflammation. C57BL/6 mice were fed HFD to induce MetS/NAFLD and orally inoculated with Pg to induce periodontitis. Part of the mice were also treated with imipramine. At the end of the study, the livers of the mice were dissected, sectioned, and subjected to F4/80 immunostaining. (**A**) Representative images of liver sections with F4/80 immunostaining. (**B**) The positively stained areas of F4/80 immunostaining were quantified and compared among all groups. Scale bar = 100 μm. The data are presented as means ± SD (*n* = 12).

To assess the effect of *P. gingivalis* inoculation, HFD feeding, and imipramine on hepatic inflammation, we performed immunohistochemical staining to detect F4/80, a biomarker for murine macrophages [44]. Results (Figure 7A,B) showed that either *P. gingivalis* inoculation or HFD feeding significantly increased F4/80 expression, and the combination of *P. gingivalis* inoculation and HFD feeding further increased its expression. In contrast, imipramine treatment significantly reduced F4/80 expression. 

### 2.6. P. gingivalis Inoculation with HFD Feeding Increases, but Imipramine Attenuates Hepatic Fibrosis

**Figure 8:**
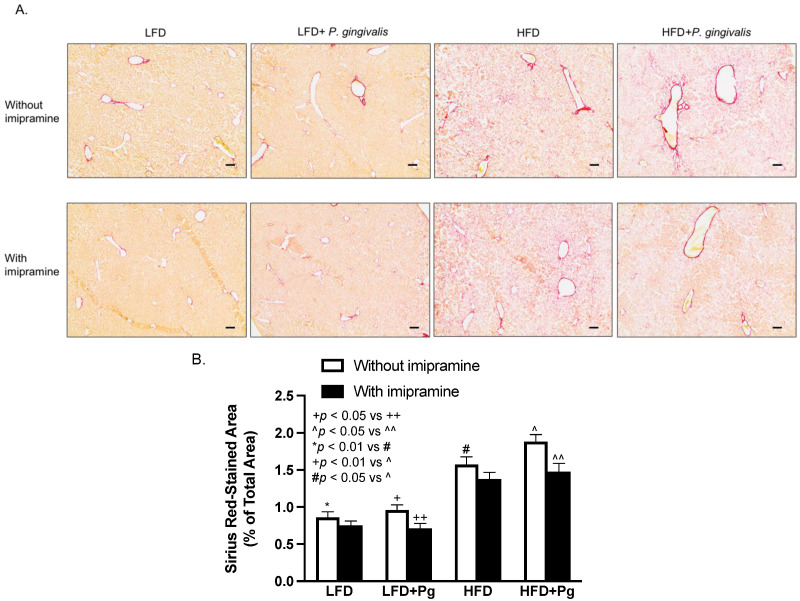
The effect of HFD feeding, oral *P. gingivalis* (Pg) inoculation, and imipramine treatment on hepatic fibrosis. C57BL/6 mice were fed HFD to induce MetS/NAFLD and orally inoculated with Pg to induce periodontitis. Part of the mice were also treated with imipramine. At the end of the study, the livers of the mice were dissected, sectioned, and stained with Sirius Red. (**A**) Representative images of liver sections with Sirius Red staining. (**B**) The positively stained areas of Sirius Red staining were quantified and compared among all groups. Scale bar = 100 μm. The data are presented as means ± SD (*n* = 12).

In addition to hepatic steatosis and inflammation, we also determined the effect of *P. gingivalis* inoculation, HFD feeding, and imipramine on hepatic fibrosis. Results from Sirius Red staining of liver sections showed that HFD feeding, but not *P. gingivalis* inoculation, markedly increased collagen content in liver sections, and the combination of *P. gingivalis* inoculation and HFD feeding further increased liver collagen content (Figure 8A,B). Contrarily, imipramine treatment significantly reversed the stimulatory effect of *P. gingivalis* inoculation and HFD feeding on hepatic fibrosis (Figure 8A,B). 

### 2.7. The Effects of P. gingivalis Inoculation, HFD Feeding, and Imipramine on the Liver Expression of Genes Involved in Lipogenesis, Inflammation, and Fibrosis 

**Figure 9:**
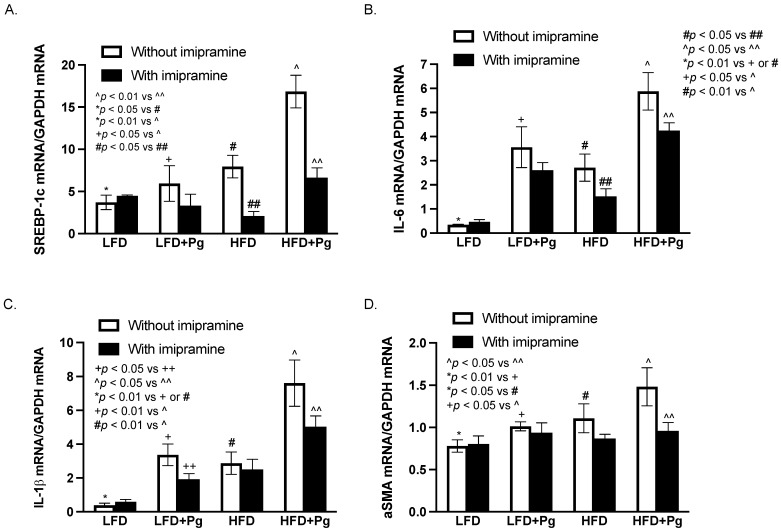
The effect of HFD feeding, oral *P. gingivalis* (Pg) inoculation, and imipramine treatment on hepatic expresScheme 57. BL/6 mice were fed HFD for 20 weeks to induce MetS/NAFLD and orally inoculated with Pg to induce periodontitis, RNA was isolated from the livers of all mice and the following mRNA was quantified using real-time PCR and normalized to GAPDH mRNA: (**A**) Sterol regulatory element-binding proteins (SREBP)-1c; (**B**) IL-6; (**C**) IL-1b; (**D**) alpha-smooth muscle actin (aSMA). The data are presented as means ± SD (*n* = 12).

To understand the molecular mechanism underlying the effects of *P. gingivalis* inoculation, HFD feeding, and imipramine on NAFLD progression and hepatic fibrosis, we quantified the expression of several hepatic genes involved in lipogenesis, inflammation, and fibrosis. Results (Figure 9A) showed that HFD feeding increased the expression of sterol regulatory element-binding protein (SREBP)-1c, a key lipogenic transcription factor [45], and the addition of *P. gingivalis* inoculation to HFD feeding further increased its expression. In contrast, imipramine significantly inhibited its expression. Results also showed that either *P. gingivalis* inoculation or HFD feeding stimulated hepatic IL-6 and IL-1b expression, and the combination of *P. gingivalis* inoculation and HFD feeding further increased the expression (Figure 9B,C). In contrast, treatment with imipramine inhibited IL-6 and IL-1b expression. Furthermore, *P. gingivalis* inoculation and HFD feeding cooperatively increased the expression of alpha-smooth muscle actin (aSMA), a key molecule involved in hepatic fibrosis [46,47], but imipramine inhibited its expression (Figure 9D). 

### 2.8. LPS and Palmitic Acid (PA) Synergistically Increase Ceramide in Macrophages In Vitro

**Figure 10:**
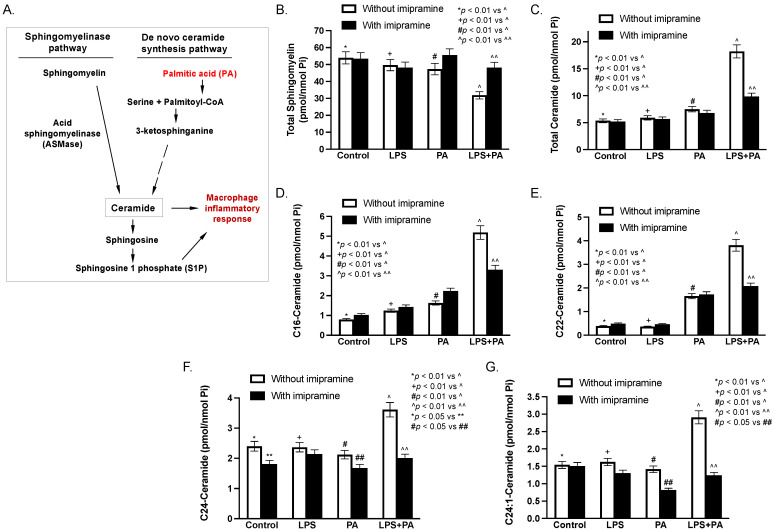
The effect of LPS, PA, LPS plus PA, and imipramine on ceramide and sphingomyelin contents in macrophages. The major sphingolipid pathways for ceramide generation are shown (**A**). RAW264.7 macrophages were stimulated with 1 ng/mL of LPS and/or 100 μM of PA for 24 h in the absence or presence of 50 mM of imipramine. After the treatments, cellular-total sphingomyelin (**B**), total ceramide (**C**), and several major ceramide species (**D**–**G**) were quantified using Lipidomics as described in Methods. The data are means ± SD (*n* = 2).

As shown in Figure 10A, ceramide can be generated from both the sphingomyelinase pathway and the de novo ceramide synthesis pathway. ASMase catalyzes the cleavage of the phosphocholine head group of sphingomyelins to generate ceramide and S1P, both of which are well-established bioactive sphingolipids playing an important role in the inflammatory response [48,49,50]. In the de novo ceramide synthesis pathway, PA is converted to palmitoyl-CoA, which conjugates with serine to form 3-ketosphinganine and subsequent dihydroceramide and ceramide [51]. 

The above results in Figure 3, Figure 4, Figure 5, Figure 6, Figure 7 and Figure 8 showed that imipramine effectively inhibited the detrimental effect of *P. gingivalis* inoculation and/or HFD feeding on periodontitis and NAFLD progression. To demonstrate how imipramine affects sphingolipid metabolism, we determined the effect of imipramine on ceramide production in macrophages in vitro since it is well-known that macrophages play a crucial role in the pathogenesis of both periodontitis and NASH [52,53,54,55]. Results showed that while either LPS or PA slightly reduced total sphingomyelin, the combination of LPS and PA markedly reduced it (Figure 10B and Table 2), confirming our previous report that LPS and PA synergistically promote sphingomyelin hydrolysis [36]. In contrast, imipramine, as an inhibitor of ASMase, inhibited the effect of LPS plus PA on sphingomyelin hydrolysis. Consistently, results showed that LPS and PA synergistically increased total cellular ceramide content, but imipramine reduced total ceramide content increased by LPS plus PA (Figure 10C and Table 3). Similar synergistic effects of LPS and PA and the inhibitory effect of imipramine were also observed on C16-, C22-, C24-, and C24:1-ceramide (Figure 10D–G and Table 3).

## 3. Discussion

In the current study, we induced both periodontitis and NAFLD in the same mice and determined if the presence of periodontitis affects the progression of MetS-induced NAFLD. We also used this animal model to determine whether targeting ASMase with imipramine will reverse the effect of periodontitis on NAFLD progression. Our data showed that the presence of periodontitis increased insulin resistance and hepatic inflammation and worsened NAFLD. When ASMase was inhibited by imipramine, insulin resistance, and hepatic inflammation were significantly reduced, and progression of NAFLD was markedly attenuated.

As expected, our metabolic study showed that while MetS elevated serum insulin levels and promoted insulin resistance, the presence of periodontitis further contributed to hyperinsulinemia and to enhanced insulin resistance. This finding is consistent with the previous clinical studies that patients with severe periodontitis have significantly increased insulin resistance [56,57]. The association between periodontitis and insulin resistance is also supported by the clinical findings that patients who received non-surgical periodontal (NSPT) therapy for periodontitis had a significant improvement in insulin resistance as compared with patients who did not receive NSPT [58].

It has been well established that hyperinsulinemia and insulin resistance enhance hepatic fat accumulation by increasing free fatty acid delivery to the liver and de novo lipogenesis and reducing fatty acid oxidation [59]. Therefore, our finding that the presence of periodontitis increases insulin resistance in mice may partially explain how the presence of periodontitis worsens NAFLD. In fact, the treatment of insulin resistance is a therapeutic approach for NAFLD, and insulin-sensitizing drugs such as thiazolidinediones have been prescribed to patients with NAFLD [60].

This study has also shown that, in addition to insulin resistance, *P. gingivalis*-induced periodontitis leads to hepatic inflammation, as evidenced by increased macrophage content and upregulation of proinflammatory cytokine expression in the liver. While both the presence of periodontitis and MetS are able to promote increased infiltration of macrophages into the liver, our data clearly show that the combination of both processes will further enhance macrophage infiltration. Similarly, the hepatic expression of proinflammatory cytokines IL-6 and IL-1b observed in MetS and periodontitis are further enhanced when both processes occur simultaneously. Given the crucial role of proinflammatory cytokines in NAFLD progression from hepatic steatosis to NASH [61], these results may unravel another mechanism by which periodontitis worsens NAFLD.

Previously, we have found that inhibiting ASMase improved MetS-exacerbated NAFLD and periodontitis in animal mode [39,40,62], suggesting that ASMase-regulated sphingolipid metabolism plays an essential role in the pathogenesis of both MetS-associated NAFLD and periodontitis. Based on the above observations, we decided to test our hypothesis that periodontitis interacts with NAFLD through ASMase-related sphingolipid metabolism. Our present study confirmed that the presence of periodontitis promoted insulin resistance and hepatic inflammation, and targeting ASMase with imipramine-reduced insulin resistance and hepatic inflammation. Given the importance of insulin resistance and hepatic inflammation in the progression of NAFLD, our findings may have uncovered a new therapeutic target to reduce or stop the progression of NAFLD. Our finding not only provided strong evidence that ASMase-regulated sphingolipid metabolism plays a crucial role in the progression of NAFLD and periodontitis but also suggests that periodontitis worsens NAFLD via sphingolipid metabolism-related insulin resistance and hepatic inflammation in mice with MetS.

**Figure 11:**
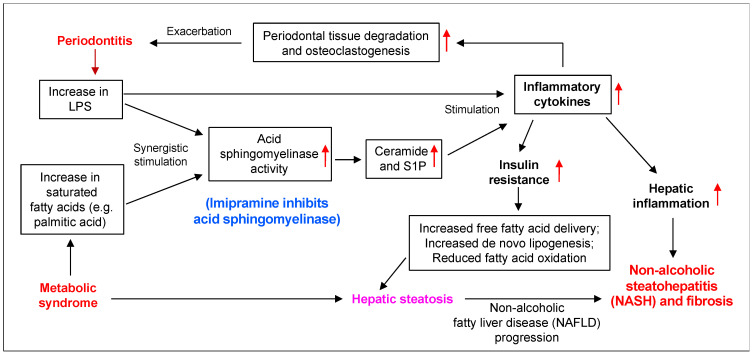
The proposed mechanisms by which periodontitis promotes HFD-induced NAFLD progression and imipramine inhibits both periodontitis and NASH in mice with MetS. Our current and previous studies have shown that periodontitis-associated LPS and MetS-related SFA, such as PA, synergistically stimulated ASMase activity in macrophages, leading to increased production of ceramide and S1P and subsequent upregulation of inflammatory cytokines. It is well known that inflammatory cytokines induce insulin resistance and hepatic inflammation, which contribute to NAFLD progression. It is also well known that inflammatory cytokines stimulate matrix metalloproteinases and osteoclastogenesis, which increase periodontal tissue degradation and alveolar bone loss. Therefore, periodontitis promotes NAFLD progression via insulin resistance and hepatic inflammation, but inhibiting ASMase by imipramine reduces ceramide and S1P production and subsequently inhibits the inflammatory cytokine expression, which improves both NAFLD and periodontitis.

As summarized in Figure 11, our previous studies [36,38] and our present study have shown that periodontitis-associated LPS and MetS-associated SFA, such as PA, synergistically stimulate ASMase, leading to increased production of ceramide in macrophages and subsequent upregulation of inflammatory cytokines such as IL-6 and IL-1b. It is well known that inflammatory cytokines promote insulin resistance [63] and hepatic inflammation [61,64], which aggravate hepatic steatosis and inflammation leading to NAFLD progression from steatosis to NASH [61]. It is also well known that inflammatory cytokines stimulate matrix metalloproteinase expression and osteoclastogenesis, promoting further periodontal tissue degradation and alveolar bone loss [65]. As a result of increased periodontal inflammation, periodontitis is likely to produce more LPS in the circulation and liver, which further worsens NAFLD.

In our investigation of how sphingolipid metabolism is dysregulated by MetS-associated NAFLD and periodontitis, we focused on macrophages since it is well-known that macrophages play a central role in both NAFLD and periodontitis [37,53,66]. Our study showed that LPS and PA exerted a robust synergy on the production of ceramide in macrophages. Since LPS and PA are associated with both innate immunity and metabolic disorders, respectively [67,68], this finding may have uncovered novel immuno-metabolic crosstalk in macrophages that are likely involved in the dysregulation of sphingolipid metabolism, resulting in increased ceramide and S1P. Furthermore, since it has been well established that ceramide is a key bioactive lipid in inducing hepatic steatosis, inflammation, and fibrosis [48,49], the stimulation of ceramide production by the crosstalk between LPS and PA is likely to be involved in the exacerbation of NAFLD by the presence of periodontitis.

It has been well established that ceramide molecules generate ceramide-enriched platforms that participate in the organization of receptors and the amplification of signaling molecules [69]. Interestingly, it has been further reported that ceramides with different acyl chains exert very different effects on the physiology and cell membrane structure [70,71]. Hammerschmidt et al. showed that C16:0 ceramide in the liver and C18:0 ceramide in skeletal muscle evolved as critical regulators of metabolic integrity and dysfunction in obesity [70], while Gupta et al. reported that C24:0 and C16:0 ceramides uniquely alter membrane dynamics by promoting the formation of cholesterol-independent domains and by elevating the inter-leaflet coupling [71].

Taken together, the findings from this study showed for the first time that the presence of periodontitis worsens NAFLD potentially through sphingolipid-mediated insulin resistance and hepatic inflammation in mice with MetS. The findings also suggest that targeting ASMase is a potentially effective approach to treat not only NASH but also periodontitis in patients with MetS.

## 4. Materials and Methods

### 4.1. Animal Feeding and Treatments

Six-week-old male C57BL/6 mice were purchased from Taconic Farms (Hudson, NY, USA) and randomly divided into eight groups (*n* = 12): (1) Low-fat diet (LFD) feeding; (2) LFD feeding and imipramine treatment; (3) LFD feeding and oral *P. gingivalis* inoculation; (4) LFD feeding, oral *P. gingivalis* inoculation, and imipramine treatment; (5) HFD feeding; (6) HFD feeding and imipramine treatment; (7) HFD feeding and oral *P. gingivalis* inoculation; (8) HFD feeding, oral *P. gingivalis* inoculation and imipramine treatment. As shown in Figure 1, MetS was induced by feeding mice HFD (D12492, 60 kcal% fat, Research Diets, Inc., New Brunswick, NJ, USA) for 20 weeks. Mice fed LFD (D12450B, 10 kcal% fat) served as controls. The amounts of fatty acids, SFAs, and PA in the HFD D12492 are 5.8, 8.2, and 42.1-fold higher, respectively, than those in the LFD D12450B diet (Table 4). All mice were housed with a 12 h light/12 h dark cycle and had free access to tap water and food. Five weeks before the completion of LFD or HFD feeding, periodontitis was induced by oral *P. gingivalis* inoculation (see the detailed inoculation procedure below). Imipramine (Sigma, St. Louis, MO, USA) at 10 mg/g body weight was given to mice by daily intraperitoneal injection in the last eight weeks (Figure 1). The Institutional Animal Care and Use Committee at the Medical University of South Carolina approved all experimental protocols. All animal-related work was performed in accordance with ARRIVE guidelines for preclinical studies and the National Institute of Health Guidelines.

### 4.2. Bacterial Culture

The *P. gingivalis* ATCC strain 33277 was purchased from American Type Culture Collection (ATCC, Manassas, VA, USA) and cultured under anaerobic conditions in Trypticase soy broth supplemented with yeast extract (1 μg/mL), menadione (1 μg/mL), and hemin (5 μg/mL) at 37 °C and harvested as described previously [72]. Bacteria for inoculation was determined at the mid-log phase [73]. All the experiments were performed in our Biosafety Level 2 laboratory, approved by the institutional biosafety committee at the Medical University of South Carolina.

### 4.3. Induction of Periodontitis by Oral P. gingivalis Inoculation

Mice were allowed to acclimate to the environment for one week before the start of the experimental procedures. Before oral inoculation of *P. gingivalis*, mice were given sulfamethoxazole (700 μg/mL) and trimethoprim (400 μg/mL) in water bottles for seven days to reduce endogenous oral flora. Normal water was given to mice for 24 h to wash out any residual antibiotics, and oral inoculation of *P. gingivalis* was performed every other day (i.e., two-day intervals) for a total of four times (i.e., at day 1, 3, 5, and 7) in a week with 10^9^ colony-forming units (CFUs) in 100 μL of PBS containing 2% carboxymethylcellulose (CMC) [74]. The sample slurry was placed directly in the oral cavity of the mice via a ball-ended 2.25 mm feeding needle [75,76]. Sham controls received vehicle 2% CMC in PBS alone. Non-infected mice and infected mice were kept in separate cages. Mice were euthanized four weeks after the last *P. gingivalis* inoculation.

### 4.4. Measurements of Metabolic Parameters

Blood samples were obtained under the fasted condition, and glucose level was determined using a Precision QID glucometer (MediSense Inc., Bedford, MA, USA). Serum cholesterol was measured using Cholestech LDX Lipid Monitoring System (Fisher Scientific, Pittsburgh, PA, USA). Serum triglycerides were measured using EnzyChrom™ Triglyceride Assay Kit (BioAssay systems, Hayward, CA, USA). Serum FFAs were determined using the EnzyChrom™ free fatty acid kit (BioAssay systems). Serum fasting insulin was assayed using the Ultra Sensitive Insulin ELISA Kit (Crystal Chem, Inc., Downers Grove, IL, USA). Fasting whole-body insulin sensitivity was estimated with the HOMA-IR according to the formula [fasting plasma glucose (mg/dL) × fasting plasma insulin (mU/mL)]/405. ALT was assayed using an ALT Assay Kit (Cayman Chemical, Ann Arbor, MI, USA).

### 4.5. Micro-CT (μCT),BVF Analysis, and CEJ-ABC Distance Measurement

Maxillae were fixed in 10% phosphate-buffered formalin for 24 h, washed with PBS, and stored in 70% ethanol. Maxillae were scanned at 55 kVp, 145 uA, 16 um voxel resolution using Scanco Medical 40 mCT scanner (Scanco Medical, Brüttisellen, Switzerland) as described previously [77]. Three-dimensional images were generated and reconstructed for each specimen. These images were rotated with a standard orientation and threshold to discern mineralized and non-mineralized tissue. The region of interest (ROI) was indicated by the contour height of molars at the cementoenamel junction as the width and the molar cusp tips to root apices as the height. Depth was equal to the buccolingual size of the teeth plus 1.0 mm^3^. Bone volume fraction (BVF) was calculated as the percentage of bone within the ROI using AnalyzePro software (Seattle, WA, USA). A well-trained and experienced research specialist who was blinded to the treatment groups performed the mCT analysis. Data are reported in accordance with standardized nomenclature [78]. Additionally, three line measurements of the distance from CEJ to ABC were taken for the first and second molars, as previously described [42].

### 4.6. Histological Tissue Processing and Pathological Evaluation

The above paraffin-embedded sections in a bucco-palatal orientation were stained with a Harris-modified H&E solution (Sigma, St. Louis, MO, USA) for evaluation of periodontal architecture and inflammatory status in the area of the periodontal ligament, teeth, and alveolar bones. The evaluation was conducted according to the following criteria for scoring tissue inflammation and bone resorption: 0 = within normal limits; 1 = focal some leukocyte infiltration, no significant bone resorption; 2 = moderate leukocyte infiltration with mild bone resorption; 3 = severe leukocyte infiltration with moderate bone resorption; and 4 = severe leukocyte infiltration with extensive bone resorption [43].

### 4.7. Histological Examination of Liver Tissue

The liver tissues were embedded in Tissue-Tek OCT compound (EMS, Hatfield, PA, USA), immediately frozen on dry ice, and stored at −80 °C. The tissue with 6 mm thickness was sectioned and mounted on glass microscope slides. The sections were fixed in 10% formalin and stained with a Harris-modified H&E solution. Slides were dehydrated and mounted in Cytoseal-XYL mounting medium (Thermo Fisher Scientific, Waltham, MA, USA). Photomicrographs of tissue sections were taken using an Olympus BX53 digital microscope with Cellsens digital image software (Olympus American Inc., Center Valley, PA, USA).

### 4.8. Oil Red O Staining

For Oil Red O staining, the frozen sections were fixed with 10% formalin for 10 min, placed in 60% isopropyl alcohol, and stained in 0.5% Oil Red O solution for 10 min. The slides were transferred to 60% isopropyl alcohol, rinsed in distilled water, and processed for hematoxylin counterstaining. Photomicrographs of tissue sections were taken using an Olympus BX53 digital microscope, and the positively stained area was quantified with ImageJ (NIH, Bethesda, MD, USA) and presented as a percent of the total area of the examined field.

### 4.9. F4/80 Immunostaining

The above-frozen sections were also subjected to immunohistochemical analysis. Liver tissues were fixed in 4% paraformaldehyde for 10 min, and frozen sections were made using a cryostat. Immunohistochemical analysis with anti-F4/80 antibodies (Cat. No. MCA497, Bio-Rad Laboratories, Inc., Hercules, CA, USA) was performed as described previously (25). Counterstaining was performed with hematoxylin. Photomicrographs of tissue sections were taken using an Olympus BX53 digital microscope, and the positively immunostained area was quantified with ImageJ (NIH, Bethesda, MD, USA) and presented as a percent of the total area of the examined field.

### 4.10. Sirius Red Staining

For Sirius Red staining, the sections were fixed with 10% formalin for 10 min, incubated with a 0.1% Sirius Red solution dissolved in aqueous saturated picric acid for 1 h, washed in acidified water (0.5% acetic acid), dehydrated, and mounted on slides. Photomicrographs of tissue sections were taken using an Olympus BX53 digital microscope, and the positively stained area was quantified with ImageJ and presented as a percent of the total area of the examined field.

### 4.11. RNA Isolation from Liver Tissues

Total RNA was isolated from mouse liver tissues using the RNeasy mini kit (Qiagen, Santa Clarita, CA, USA) by following the instructions provided by the company.

### 4.12. Real-Time Polymerase Chain Reaction (PCR)

Real-time PCR was performed as previously described [36]. The mouse (SREBP)-1c, IL-6, IL-1b, aSMA, and glyceraldehyde-3-phosphate dehydrogenase (GAPDH) were quantified by PCR. The primers (SREBP-1c, forward: GGAGCCATGGATTGCACATT; reverse: GGCCCGGGAAGTCACTGT); IL-6, forward: TGGAGTCACAGAAGGAGTGGCTAAG; reverse: TCTGACCACAGTGAGGAATGTCCAC; IL-1b, forward: CTGTACGATCACTGAACTGC; reverse: CACCACTTGTTGCTCCATATC; aSMA, forward: GAGGCACCACTGAACCCTAA; reverse: CATCTCCAGAGTCCAGCACA); GAPDH, forward: CTGAGTACGTCGTGGAGTC; reverse: AAATGAGCCCCAGCCTTC) were synthesized by Integrated DNA Technologies, Inc. (Coralville, IA, USA). The PCR data were analyzed with the iCycler iQ^TM^ (Bio-Rad, Hercules, CA, USA). An average starting quantity (SQ) of fluorescence units was used for analysis. Quantification was calculated using the SQ of targeted cDNA relative to that of GAPDH cDNA in the same sample.

### 4.13. Cell Culture

RAW264.7 macrophages were purchased from the American Type Culture Collection and grown in DMEM (ATCC) supplemented with 10% heat-inactivated fetal calf serum (HyClone, Logan, UT, USA). The cells were maintained in a 37 °C, 90% relative humidity, and 5% CO_2_ environment. RAW264.7 macrophages were treated with 1 ng/mL of LPS, 100 μM of PA, and 50 μM of imipramine (Sigma, St. Louis, MO, USA).

### 4.14. PA Preparation

To prepare PA for cell treatments, PA (Sigma, St. Louis, MO, USA) was dissolved in 0.1 N NaOH and 70% ethanol at 70 °C to make PA with a stock concentration of 50 mM. The solution was kept at 55 °C for 10 min, mixed, and brought to room temperature.

### 4.15. Lipidomics

RAW264.7 cells were collected, fortified with internal standards, extracted with ethyl acetate/isopropyl alcohol/water (60:30:10, *v*/*v*/*v*), evaporated to dryness, and reconstituted in 100 μL of methanol. Simultaneous ESI/MS/MS analyses of sphingoid bases, ceramides, and sphingolipids were performed on a Thermo Finnigan TSQ 7000 triple quadrupole mass spectrometer operating in multiple reactions monitoring positive ionization mode. The phosphate contents of the lipid extracts were used to normalize the MS measurements of sphingolipids. The phosphate contents of the lipid extracts were measured with a standard curve analysis and a colorimetric assay of ashed phosphate [79].

### 4.16. Statistical Analysis

GraphPad Prisma 8 (Version 8.4.3) (GraphPad Software, Inc. La Jolla, CA, USA) was used for statistical analysis. The one-way analysis of variance (ANOVA) with the post hoc test was used to determine whether there were any statistically significant differences between the means of three or more independent groups. A Student *t*-test was performed for comparison between the two groups when the data had a normal distribution. For data without normal distribution, nonparametric analysis using the Mann–Whitney test was performed. When an experiment was repeated, statistical analysis was also performed to compare their variances. The values were expressed as mean ± SD, and a value of *p* < 0.05 was considered significant.

## 5. Conclusions

In the present study, we showed that the presence of periodontitis promoted the progression of NAFLD to NASH by increasing insulin resistance and hepatic inflammation in mice. Inhibiting ASMase with imipramine-reduced insulin resistance and hepatic inflammation and improved both NASH and periodontitis. We also showed that while LPS and PA synergistically increased ceramide in macrophages, imipramine significantly reduced it. Since the role of ceramide in hepatic steatosis, inflammation, and fibrosis has been well established, this study suggests that targeting ASMase is a potential approach to reduce the contribution of periodontitis to NAFLD progression.

## Figures and Tables

**Table 1:** The Bone Volume Fraction (BVF) of Mice with or without *P. gingivalis* Inoculation, Metabolic Syndrome (MetS) and Imipramine Treatment.

	Mice Without Imipramine Treatment		Mice with Imipramine Treatment	
	BVF	% BVF Change Compared to Control Mice	BVF	% BVF Change Compared to Mice without Imipramine Treatment
Control (LFD-fed)	0.663 ± 0.010	−	0.676 ± 0.009	+1.96%
*P. gingivalis* inoculation	0.626 ± 0.009	−5.58% *	0.679 ± 0.007	+8.47% **
MetS (HFD-fed)	0.624 ± 0.009	−5.88% **	0.621 ± 0.010	−0.48%
MetS + *P. gingivalis* inoculation	0.587 ± 0.008	−11.46% **	0.631 ± 0.008	+7.50% **

The BVF was quantified as described in Materials and Methods. In mice without imipramine treatment, the BVF for mice treated with *P. gingivalis* inoculation and/or HFD was compared with that for control mice (LFD-fed alone). In mice with imipramine treatment, the BVF in each group was compared with that in mice without imipramine treatment. * *p* < 0.05; ** *p* < 0.01.

**Table 2:** LPS and PA Synergistically Increase Sphingomyeline (SM) Hydrolysis but Imipramine (IMP) Robustly Attenuates It in Macrophages.

	C16-SM	C18-SM	C20-SM	C22-SM	C24-SM	C24:1-SM	Total-SM
Control	34.09	2.13	0.71	3.65	2.46	6.64	53.70
Control + IMP	34.13	2.30	0.77	3.82	2.40	6.21	53.46
LPS	33.40	1.55	0.55	2.74	2.02	5.16	49.59
LPS + IMP	32.24	1.89	0.61	3.04	2.39	4.62	48.22
PA	26.55	4.23	1.33	5.98	2.54	6.87	50.67
PA + IMP	33.29	3.89	1.08	4.99	2.25	6.76	55.54
LPS + PA	18.84	2.20	0.64	2.75	1.15	4.16	31.80
LPS + P + IMP	30.63	2.76	0.81	3.52	1.58	5.84	48.14

RAW264.7 macrophages were treated with 1 ng/mL of LPS, 100 μM of PA or both in the absence or presence of 50 μM of imipramine for 24 h. The cells are collected and subjected to lipidomic analysis as described in Materials and Methods. Unit: pmol/nmol phosphate.

**Table 3:** LPS and PA Synergistically Increase Ceramide (CER) Production but Imipramine (IMP) Robustly Inhibits CER Production in Macrophages.

	C14-Cer	C16-Cer	C18-Cer	C20-Cer	C22-Cer	C24-Cer	C24:1-Cer
Control	0.79	0.05	0.03	0.39	2.40	1.54	5.36
Control + IMP	1.03	0.13	0.04	0.49	1.81	1.51	5.20
LPS	1.24	0.06	0.03	0.36	2.37	1.62	5.90
LPS + IMP	1.42	0.11	0.05	0.46	2.14	1.30	5.69
PA	1.62	0.30	0.18	1.65	2.12	1.42	7.50
PA + IMP	2.23	0.49	0.24	1.73	1.68	0.81	7.32
LPS + PA	5.18	1.31	0.70	3.81	3.61	2.91	18.21
LPS + PA + IMP	3.30	0.66	0.30	2.08	2.01	1.24	9.85

RAW264.7 macrophages were treated with 1 ng/mL of LPS, 100 μM of PA or both in the absence or presence of 50 μM of imipramine for 24 h. The cells are collected and subjected to lipidomic analysis as described in Materials and Methods. Unit: pmol/nmol phosphate.

**Table 4:** The Total Fat, Fatty Acid, Saturated Fatty Acid and Palmitic Acid Contents in High-Fat Diet D12492 and Low-Fat Diet D12450B.

	Total Fat	Fatty Acid	Saturated Fatty Acid	Palmitic Acid
D12492	60% kcal fat	254.5 g/kg	81.5 g/kg	51.0 g/kg
D12450B	10% kcal fat	43.7 g/kg	9.9 g/kg	1.1 g/kg

The content of total fat was presented as percent of kcal from total fat and the contents of fatty acid, saturated fatty acid and palmitic acid were presented as gram per kg of diet.

## Data Availability

All data generated or analyzed during the current study are available from the corresponding author upon reasonable request.
